# Structure of the drug target ClpC1 unfoldase in action provides insights on antibiotic mechanism of action

**DOI:** 10.1016/j.jbc.2022.102553

**Published:** 2022-10-06

**Authors:** Katharina Weinhäupl, Marcos Gragera, M. Teresa Bueno-Carrasco, Rocío Arranz, Olga Krandor, Tatos Akopian, Raquel Soares, Eric Rubin, Jan Felix, Hugo Fraga

**Affiliations:** 1 i3S, Instituto de Investigacao e Inovacao em Saude, Universidade do Porto, Porto, Portugal; 2 Department Macromolecular Structures, Cryo-EM Facility, Centro Nacional de Biotecnología-CSIC, Madrid, Spain; 3 Department of Immunology and Infectious Diseases, Harvard Chan School of Public Health, Harvard University, Cambridge, Massachusetts, USA; 4 Departamento de Biomedicina, Faculdade de Medicina da Universidade do Porto, Porto, Portugal; 5 Unit for Structural Biology, Department of Biochemistry and Microbiology, Ghent University, Ghent, Belgium; 6 Unit for Structural Biology, VIB-UGent Center for Inflammation Research, Ghent, Belgium

**Keywords:** Mycobacterium tuberculosis, antibiotics, chaperone, protein degradation, cryo-electron microscopy

## Abstract

The unfoldase ClpC1 is one of the most exciting drug targets against tuberculosis. This AAA+ unfoldase works in cooperation with the ClpP1P2 protease and is the target of at least four natural product antibiotics: cyclomarin, ecumicin, lassomycin, and rufomycin. Although these molecules are promising starting points for drug development, their mechanisms of action remain largely unknown. Taking advantage of a middle domain mutant, we determined the first structure of *Mycobacterium tuberculosis* ClpC1 in its apo, cyclomarin-, and ecumicin-bound states via cryo-EM. The obtained structure displays features observed in other members of the AAA+ family and provides a map for further drug development. While the apo and cyclomarin-bound structures are indistinguishable and have N-terminal domains that are invisible in their respective EM maps, around half of the ecumicin-bound ClpC1 particles display three of their six N-terminal domains in an extended conformation. Our structural observations suggest a mechanism where ecumicin functions by mimicking substrate binding, leading to ATPase activation and changes in protein degradation profile.

Clp proteases are composed of two heptameric rings, forming a cylinder with 14 proteolytic sites compartmentalized within its central chamber (1). While ClpP alone is able to rapidly hydrolyze peptides, the degradation of large proteins requires the presence of a hexameric AAA+ ATPase complex, such as ClpX or ClpC1. These ATPases activate the Clp proteases but also bind protein substrates, unfold them, and translocate them into the proteolytic compartment. Unlike most bacteria and mitochondria, *Mycobacterium tuberculosis* (*Mtb*) contains two clp genes, clpP1 and clpP2, that form an active complex containing one ClpP1 and one ClpP2 ring (unless otherwise stated, ClpP1P2, ClpC1, and ClpX refer to *Mtb* proteins) (2, 3). In addition to genetic evidence that ClpC1, ClpX, ClpP1, and ClpP2 proteins are essential for viability, the relevance of these targets has been reinforced by the discovery of multiple natural product antibiotics (NPAs) that kill *Mtb* by targeting this system. The specific potential of ClpC1 as a drug target in *Mtb* was proven by the discovery of four potent and chemically diverse NPAs acting on this protein (4, 5, 6, 7, 8, 9, 10). Indeed, the cyclic peptides ecumicin, cyclomarin, rufomycin, and lassomycin, all binding ClpC1, are among the most powerful anti-tuberculosis (TB) molecules to emerge recently. Ecumicin, for example, displays potent selective anti-TB activity with an MIC value 50 times lower than that of rifampicin or isoniazid, the first line drugs for the treatment of TB (4, 5).

ClpC1 is a member of the class II AAA+ family of proteins, which contains an N-terminal domain (NTD) and two distinct ATP-binding modules, D1 and D2. While no full-length structure of *Mtb*ClpC1 is currently available, considerable structural work has been performed on the easy-to-handle NTD domain (7, 11, 12). Curiously, despite representing only a small portion of the full protein, all the NPAs have been shown to bind to the ClpC1-NTD domain, and high-resolution X-ray structures of the binding sites are available for cyclomarin, ecumicin, and rufomycin (7, 11, 12).

While this allows a proper mapping of the NPA-binding pockets, it is still not clear how binding to the NTD can translate into functional impairment of the remaining protein. The NTD lacks ATPase activity, which is present in the D1 and D2 domains, and is connected to these domains by a long, disordered loop. Somehow, binding to the NTD must induce changes in the remaining domains. Curiously, despite binding to similar parts of the NTD, the different NPAs lead to distinct effects.

Using small-angle X-ray scattering, we have recently shown that ClpC1 exists in an equilibrium between a resting state and the active hexameric state, but how this equilibrium is modulated in *Mtb* is still difficult to understand (7). MecA, an adapter that modulates ClpC in other species, does not exist in the *Mtb* genome, but a homolog of the ClpS adaptor protein has been shown to bind to ClpC1 (13). Another possibility is that substrate binding, as proposed by others, can shift the ClpC1 equilibrium towards a hexamer (14). It is assumed, based on the extensive AAA+ literature and obtained structural data, that the hexameric state is the ClpC1 functional form. Indeed, only the hexameric form permits the formation of a surface for the interaction with ClpP as well as a pore linked with loops that can couple ATP hydrolysis to mechanical substrate pulling. It is rather unlikely that the structure observed for the resting state, despite the low resolution, can interact with ClpP.

Taking advantage of a single mutation, we were able to stabilize the hexameric state of this important drug target and obtain structures in the presence of cyclomarin and ecumicin. Interestingly, half of the ecumicin-bound particles display three of their six NTDs in an extended conformation, suggesting a potential mechanism of how ecumicin modulates ClpC1 function.

## Results

### Mutation of phenylalanine 444 into alanine results in a fully functional *Mtb*ClpC1 with a stabilized hexameric state

Although the ClpC1-NTD has been extensively studied by X-ray crystallography and solution NMR (7, 11, 12, 15), no structural information on the full-length ClpC1 has so far been reported. This can be rationalized by several key factors. Firstly, we have shown that ClpC1 exists in an equilibrium between a resting state and the active hexameric state (7). Second, the active hexamer is only formed in the presence of ATP, which is rapidly converted into ADP. Finally, the ClpC1P1P2 *Mtb* system is, compared to homologs from *Staphylococcus aureus* and *Bacillus subtilis*, more insoluble and therefore harder to work with *in vitro* (7, 16). Indeed, these limitations and the fact that so far all the NPAs targeting ClpC1 bind to the ClpC1 NTD have led others to employ chimeras of ClpC1 and the D1D2 domains of *S. aureus* to study the function and mode of action of cyclomarin (16). While this is an interesting approach, it has the limitation of inferring results from nonphysiological proteins and therefore not providing any direct and useful structural data for drug development. This is particularly evident, considering the existing mechanistic differences between the *S. aureus* and *Mtb* proteins. For example, whereas *Sa*ClpC depends on MecA to catalyze the degradation of GFPssra by ClpP, ClpC1 is fully capable of doing so, independently of the adapter (2, 17, 18, 19). In fact, MecA does not exist in the *Mtb* genome. It is therefore fundamental to obtain structural information on ClpC1 in its functional state.

**Figure 1:**
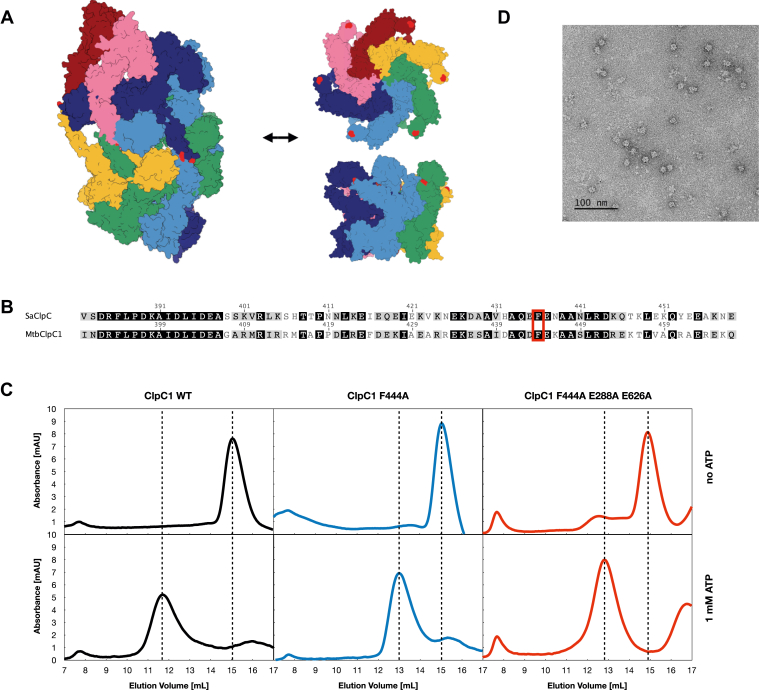
**ClpC1 resting state – hexamer equilibrium.***A*, model of the *Mtb*ClpC1 resting state created by Swiss-model using the *Staphylococcus aureus* ClpC resting state as a template (PDB: 6EM9) and of the *Mtb*ClpC1 theoretical active hexameric state using the crystal structure of *Bacillus subtilis* ClpC (PDB 3PXI). The protomers P1 to P6 are shown in *rainbow* colors. P1, *pink*; P2, *dark blue*; P3, *light blue*; P4, *green*; P5, *yellow*; and P6, *dark red*. Residue F444 is marked in *light red*. *B*, extract of the pairwise sequence alignment of *Mtb*ClpC1 and *S. aureus* ClpC. The conserved F444 residue is marked in *red*. *C*, size exclusion profile of WT *Mtb*ClpC1 (*black*) and the two mutants *Mtb*ClpC1 F444A (*blue*) and *Mtb*ClpC1 F444A E288A E626A (*red*) in the absence and presence of ATP. *D*, negative stain EM images of the hexameric ClpC1 F444A mutant. Mtb, Mycobacterium tuberculosis; NPAs, natural product antibiotics; PDB, Protein Data Bank.

Given the presence of an equilibrium between a decameric resting state and the active hexameric ClpC1 states in solution, we rationalized that the stabilization of the ClpC1 hexamer could allow us to obtain structural information on this important drug target. Previously, stabilization was achieved by removing intrinsically disordered loops, allowing the elucidation of the only ClpC X-ray structure described to date (20), but at the expense of enzymatic activity and therefore mechanistic relevance. Carroni *et al.* (14) have demonstrated that point mutations in the middle domain (MD) of *Sa*ClpC shift the equilibrium towards the hexameric state. We hypothesized that similar modifications could also stabilize the active hexameric state of ClpC1, allowing subsequent structural characterization. In particular, residue F436 in *Sa*ClpC (F444 in *Mtb*) has been shown to be important for the resting state–hexamer equilibrium (Figs. 1, *A* and *B* and S2*A*).

By means of site-directed mutagenesis, we mutated the homologous residue in ClpC1 to an alanine (F444A), to test if it could shift the ClpC1 equilibrium. Using a Superose 6 10/300 GL column, we observed that, as previously shown (7), WT ClpC1 (ClpC1_WT_) migrates as a species larger than the canonical hexamer. By contrast, in the presence of ATP, the F444A main species (ClpC1_F444A_) eluted in a volume consistent with the size expected for a ClpC1 hexamer (Fig. 1*C*). Furthermore, the presence of hexameric rings in the mutant was also confirmed by negative stain EM (Fig. 1*D*).

**Figure 2:**
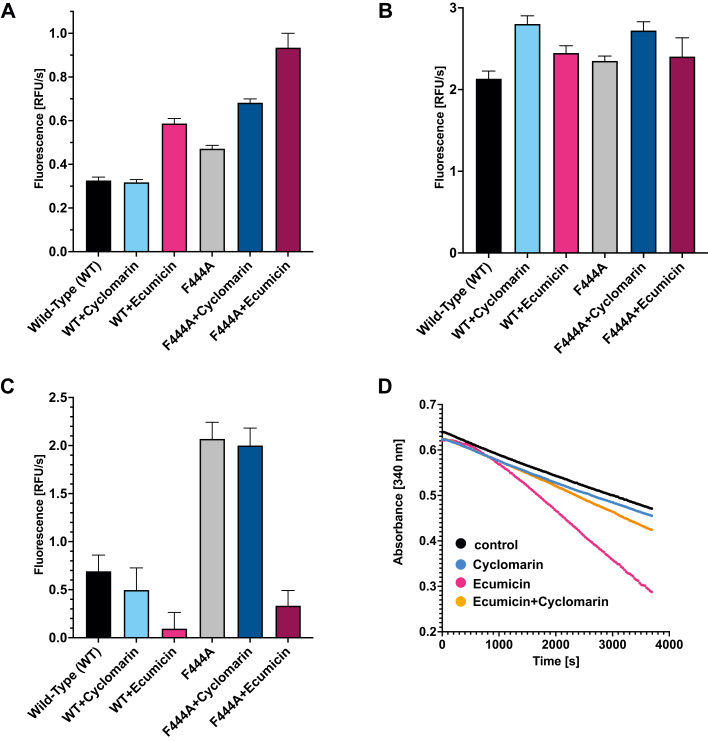
**Effect of NPAs on ClpC1 activity.***A*, ATPase activity of WT ClpC1 and the F444A mutant in the apo state and bound to natural product antibiotics. Degradation of FITC-casein (*B*) and GFPssra (*C*) by the ClpC1P1P2 complex of apo ClpC1 and the F444A mutant in the presence of natural product antibiotics. *D*, cyclomarin binding prevents the activation of ClpC1 ATPase activity promoted by ecumicin binding. NPAs, natural product antibiotics.

After successful stabilization of the hexameric state, we sought to verify that biological activity was maintained in mutant ClpC1_F444A_. ClpC1_F444A_ ATPase activity was checked using an assay monitoring the fluorescence decrease at 340 nm, associated with NADH to NAD^+^ conversion as the ADP formed by ClpC1 ATPase activity is reconverted into ATP by pyruvate kinase and phosphoenolpyruvate dehydrogenase (18). As shown in Figure 2*A*, ClpC1_F444A_ displayed an increase in the ATPase activity in comparison to ClpC1_WT_. An increase in ATPase activity was also observed for the *Sa*ClpC1_F436A_ mutant, which was explained by a shift in the resting state–active hexamer equilibrium towards the latter, which is consistent with the described size-exclusion chromatography experiments (14).

Although cyclomarin and ecumicin binding sites are located at the ClpC1 NTD and are therefore distant from the F444A mutation, we tested the functional consequences of cyclomarin and ecumicin binding on the mutant. Using saturating concentrations (10 μM), ecumicin binding to the WT results in a strong increase in ClpC1 ATPase activity, while cyclomarin does not. Curiously, a similar increase in ATPase activity is observed using the F444A mutant, showing that ecumicin effects do not exclusively result from the modulation of the ClpC1 resting state–hexamer equilibrium (Fig. 2*A*). An important *in vivo* function of ClpC1, if not the most relevant, is to associate with ClpP1P2 and target proteins for degradation by the protease. The roles of ClpC1 in this process are multiple, as it is responsible for substrate recognition, unfolding, and translocation but also for association with ClpP1P2 and its concomitant allosteric activation (2, 18). Because these processes are very hard to study independently, ClpC1 function is often evaluated indirectly by measuring protein target degradation, that is, the rate at which a protein is degraded by ClpP1P2 in association with ClpC1 (2). Two substrates that are commonly used are FITC-casein (casein with a fluorescein fluorophore attached to lysine residues) and GFP, representing two classes of proteins. FITC-Casein, which lacks a well-defined tertiary structure, is usually used as a model for unfolded substrates, while the stable, beta-barrel containing GFP is used as a model for a structured protein. While in the case of FITC-casein, protein degradation results in a net fluorescence increase as fluorescein quenching is reduced with degradation; in the case of GFPssra, degradation of the protein fluorophore results in a decrease in fluorescence (18). As can be seen in Figure 2, *B* and *C*, the F444A mutant is fully functional to catalyze the degradation of both FITC-casein and GFPssra by ClpP1P2, showing that this mutation does not impair ClpC1 enzymatic activity.

Reflecting different modes of action, the effects of cyclomarin and ecumicin in protein degradation by the ClpC1P1P2 complex are distinct. At the concentration used, cyclomarin is a moderate activator of FITC-casein degradation and a weak inhibitor of GFPssra degradation, whereas for ecumicin, we observe a mild activation of FITC-casein degradation and a strong inhibition of GFPssra degradation (Fig. 2, *B* and *C*). The different effects of these two NPAs are also clear when they are used together. Indeed, cyclomarin is able to prevent both ATPase activation (Fig .2*D*) as well as GFP degradation inhibition induced by ecumicin (Fig. S1), showing that they are competing for a similar pocket but they induce distinct biochemical effects. The distinct structural consequences resulting from cyclomarin and ecumicin binding are also clear from other biophysical data. Differential scanning fluorimetry is a very useful method to monitor ligand binding to a given target. We have previously shown that arginine phosphate (ArgP) is able to stabilize the NTD, and we aimed to test the effects of cyclomarin and ecumicin on the NTD (7). Using Sypro orange as a fluorescence reporter, we obtained a Tm of 77.7 (±0.3) °C and 83.6 (±0.3) °C, for the apo and ArgP-bound NTD. These values are higher than the ones we previously reported using intrinsic tryptophan fluorescence as a reporter that were 69 °C and 79 °C for the WT and ArgP-bound NTD (7). Quite striking was the difference observed between cyclomarin and ecumicin. While cyclomarin binding resulted in a very strong stabilization of NTD, with a calculated Tm of 91.5 (±0.9) °C, ecumicin, on the contrary, did not stabilize the domain and in fact lead to a decrease in the Tm (Fig. S2*B*).

### Structural characterization of *Mtb*ClpC1 by cryo-EM

Having shown that the MD mutant (ClpC1_F444A_) is active, we attempted unsuccessfully to crystallize the resulting hexamer. We therefore set out to obtain structural information on ClpC1 via cryo-EM. To further stabilize the sample, considering that ATP is being continuously degraded even at low temperatures and that some AAA+ proteins have been shown to hydrolyze ATP analogs, we introduced two additional mutations in the double walker B motif (E288A and E626A). We hypothesized that these mutations allow nucleotide binding but prevent hydrolysis and therefore further stabilize the hexameric assembly.

**Figure 3:**
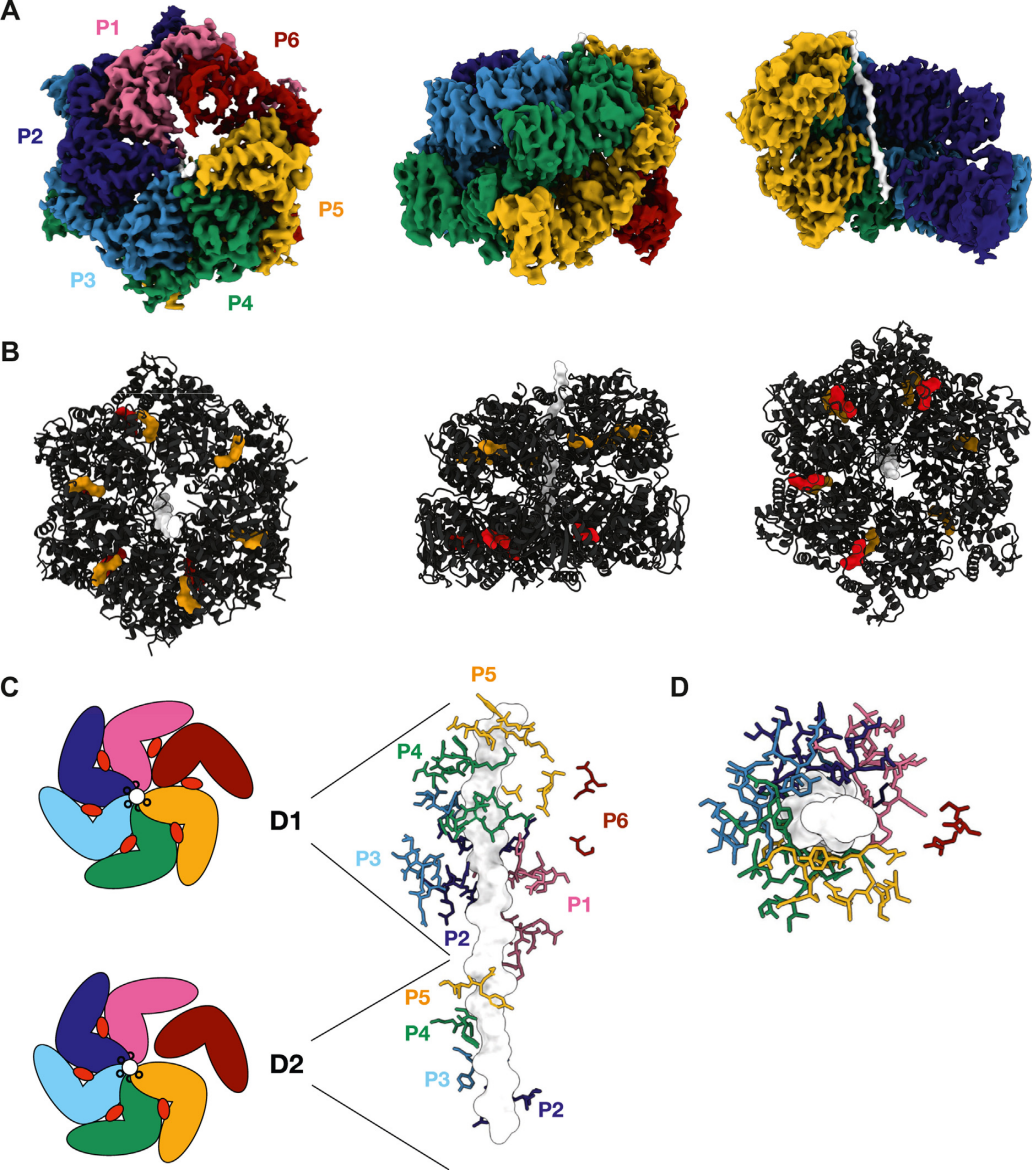
**The *Mtb*ClpC1 active hexameric structure.***A*, cryo-EM map of the apo *Mtb*ClpC1 hexamer bound to a substrate peptide in top, side view and with the bound substrate visible (without the protomers P1 and P6). Individual protomers are colored independently and labeled P1 to P6. *B*, occupation of the nucleotide-binding pockets in the *Mtb*ClpC1 hexamer with ADP (in the D1 domain *orange*, in the D2 domain *red*). From left to right hexamer in top view (substrate entry pore), side view, and bottom view (interface with ClpP1P2). *C*, cartoon image of the nucleotide occupation in the D1 and D2 domains and the attachment of the pore loops in D1 and D2 to the substrate. P6 is detached from the substrate in both D1 and D2. *D*, top view of the ClpC1-bound substrate with pore loops attached in the typical spiral arrangement and the pore loop of P6 detached. Mtb, Mycobacterium tuberculosis.

After initial cryo-EM grid screening, a dataset was collected of the apo form of *Mtb*ClpC1_E288A/F444A/E626A_ (see Experimental procedures). Processing of the data resulted in a structure for apo ClpC1 determined to a resolution of 3.6 Å (see Experimental procedures, Figs. S3 and S4, and Table S1). As expected, the overall structure of apo *Mtb*ClpC1_E288A/F444A/E626A_ consists of a hexamer composed of six ClpC1 subunits (A–F), with the D1 and D2 domains arranged in a ring that together forms a pore through which the substrate can be actively translocated. In line with recent structures, including the recent cryo-EM structure of *B. subtilis* ClpC (21), the observed ClpC1 hexamer is not symmetric, as observed originally in the crystal structure of ClpC (20), and instead adopts an asymmetric spiral structure (Fig. 3*A*). An important characteristic of our structure is the observation of a 23-residue long peptide visible in its central pore. The presence of substrates in the pore formed by D1 and D2 has been previously reported for other members of the family, but as no substrate has been added to our cryo-EM sample preparation, it was probably taken up and trapped inside of the inactive protein during purification (Fig. 3, *A* and *B*). This unexpected finding is nevertheless useful as it provides important details on the ClpC1 mechanism of action.

Indeed, the substrate is bound by each of the two pore loops from subunits A to E in both D1 and D2, with the exception of pore loop 2 in D1 subunit E, forming a spiral along the substrate. Subunit E is positioned at the highest point and subunit A at the lowest. Subunit F is detached from the substrate in both D1 and D2, and the pore loops are not visible. Furthermore, all nucleotide-binding sites in D1 are occupied by ADP, while for D2, four sites are occupied by ADP (subunits B, C, D, and E), while two are in their apo state (subunits A and F) (Fig. 3*C*). This organization is in line with the asymmetric disposition we referred to above and has been reported for other members of the family, particularly for structures obtained using cryo-EM (21, 22, 23).

Unfortunately, several structural features are not visible in the ClpC1 apo structure, presumably due to flexibility or intrinsic disorder. One of these missing features are the LGF loops which, in line with other AAA+ ATPase structures, will likely only become structured upon interaction with the ClpP protease (22). Another unresolved feature is the MD, which connects D1 and D2 and carries the F444A mutation. The MD is presumably a key point for interactions with adapters or binding partners and is usually only visible in the resting state or when it is engaged with a binding partner (see MecA in ClpC1 or DnaK in the homologous ClpB) (14, 24). Although there is no high-resolution structure of the ClpC1 resting state, we presume that the overall organization of the resting state is similar to *Sa*ClpC for two reasons: (1) our previously reported small-angle X-ray scattering structure of the ClpC1 resting state overlays perfectly with the *Sa*ClpC cryo-EM structure of the resting state (Protein Data Bank, PDB: 6EM9/6EM8) and (2) the same residues appear to be involved in the stabilization of the resting state as shown by our mutation experiments involving F444. It is thus likely that the MD of ClpC1 in the resting state is properly folded to provide contacts between the different MDs, thereby stabilizing the resting state, and only becomes unfolded upon formation of the hexamer. Finally, the NTD and its adjacent 26 residue linker are not visible in our apo ClpC1 structure. The NTD is thought to have an important role in substrate recognition and targeting to the ClpC1 pore, and it is the binding site of several recently identified NPAs against *Mtb*. Similar to the MD, the NTD can only be seen in the resting state or in structures where an adapter or the substrate itself stabilizes its position. The isolated NTD is a well-folded globular domain and the fact that it is not visible in our apo ClpC1 structure presumably does not reflect on an intrinsic disorder of the domain but rather on the high flexibility of the linker and the resulting multiple orientations with respect to the D1/D2 domains.

### Cryo-EM structures of *Mtb*ClpC1 bound to NPAs

Following successful structure determination of apo ClpC1, two additional datasets were collected on cryo-EM grids prepared after the addition of 30 μM cyclomarin or ecumicin to purified *Mtb*ClpC1_E288A/F444A/E626A_ (see Experimental procedures). Processing of the two datasets resulted in a structure for cyclomarin-bound ClpC1 determined to a resolution of 3.3 Å and two 3D classes of ecumicin-bound ClpC1 determined to resolutions of 4.3 Å and 8.6 Å, respectively (see Experimental procedures, Figs. S3 and S4, and Table S1).

The structure of cyclomarin-bound ClpC1 (Fig. 2*C*) is virtually identical to the apo ClpC1 structure, with a calculated rmsd of 0.39 Å (over 3239 aligned C_α_-atoms). No differences in nucleotide binding or substrate interaction were observed. Similar to the apo ClpC1 structure, a 23-residue substrate is trapped in the ClpC1 pore and the NTDs, MDs, and LGF loops are invisible.

**Figure 4:**
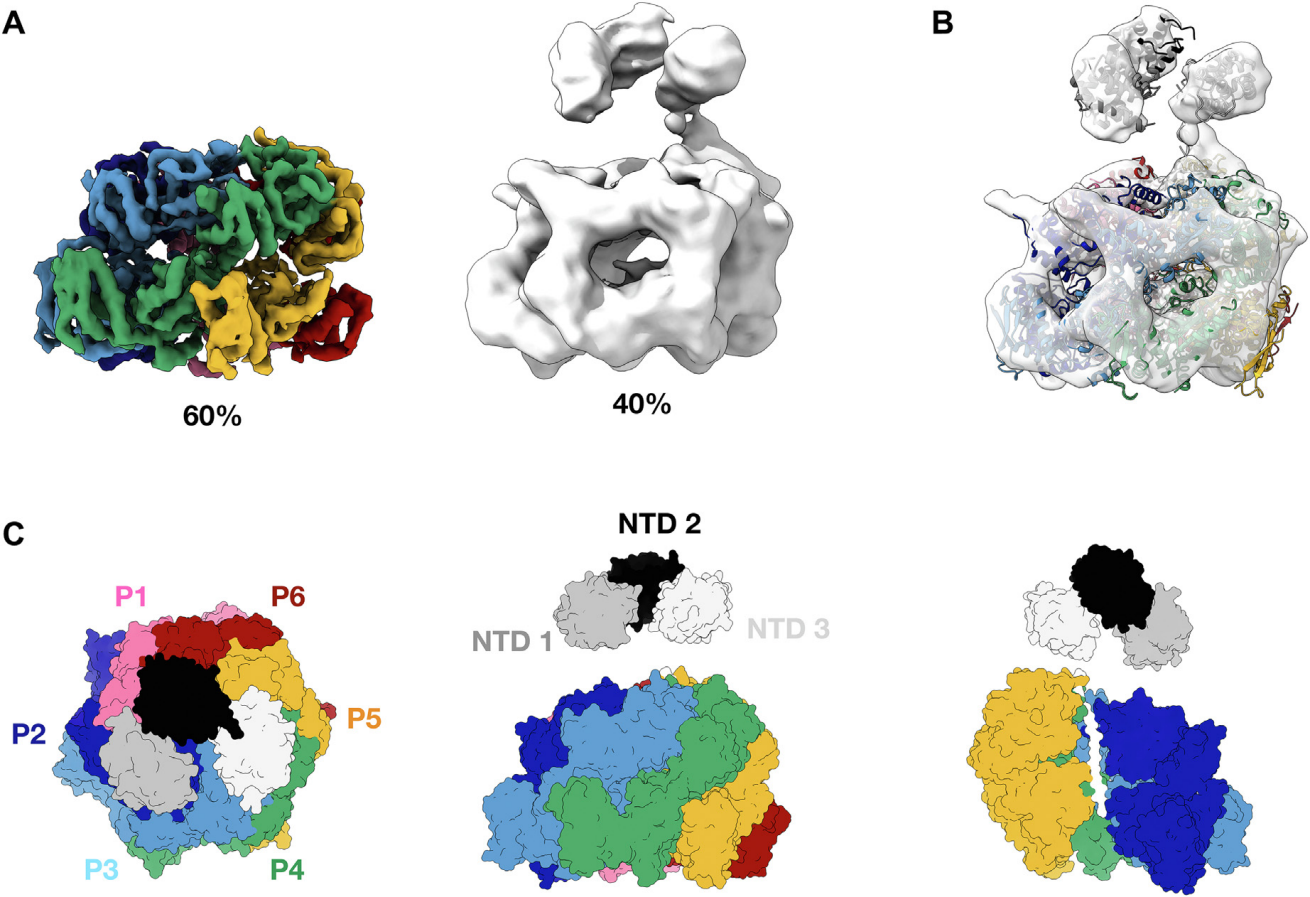
**Structural basis of the ecumicin mechanism of action.***A*, class 1 (*rainbow*) and 2 (*white*) maps of ecumicin-bound *Mtb*ClpC1 with the percentage of particles found in each state. *B*, class 2 ecumicin-bound ClpC1 map with the fitted model of the *Mtb*ClpC1 NTD (created by alpha fold) and the hexameric high-resolution structure of ecumicin-bound *Mtb*ClpC1 (*rainbow cartoon*). *C*, model of the ecumicin-bound hexameric ClpC1 and three visible NTD domains in top, side, and substrate visible view (without P1 and P6). Protomers P1 to P6 are colored individually, because of the low-resolution map and the invisibility of the linker region, NTDs cannot be assigned to specific protomers and are therefore colored in grayscale. Mtb, Mycobacterium tuberculosis; NTD, N-terminal domain.

Processing of the *Mtb*ClpC1 dataset with added ecumicin resulted in 2D classes that included side-views revealing hints of the NTDs, present as a blurry sphere on top of the ClpC1 hexamer (Fig. S3*A*). Ensuing hetero refinement using two 3D classes followed by final non-uniform (NU) refinements in cryoSPARC (https://cryosparc.com) (25) resulted in two distinct maps at resolutions of 4.3 Å and 8.6 Å. The first map, populated by roughly 60% of the particles, represents a structure identical to the ones observed for apo *Mtb*ClpC1 and cyclomarin-bound *Mtb*ClpC1. The second map, containing 40% of particles, has a significantly lower resolution but reveals three globular domains positioned on top of the D1 domains of the hexameric ClpC1 ring in an organization reminiscent of an NTD trimer observed in a hyperactive ClpB mutant bound to casein (26). Rigid-body fitting of the NTD-trimer taken from PDB 6OG3 (26) in the low-resolution map results in a good fit (Fig. 4*B*) and provides a model where the antibiotic-binding sites in helix 1 and 5 on the NTD and the ArgP/substrate-binding sites are facing towards the center of the ClpC1 hexameric ring (Fig. 4, *A* and *B*). The three remaining NTD domains as well as all flexible linkers connecting the NTD domains with the adjacent D1 domain are not visible on the map, most probably due to the inherent flexibility of the linker region. Nonetheless, the observed trimerization of the *Mtb*ClpC1 NTDs upon addition of ecumicin suggests that ecumicin can function by mimicking substrate binding.

## Discussion

It is well established that minor differences in protein structure may result in important functional differences. Therefore, even when structures of homologs are available, it is still important to obtain accurate structural information, particularly for drug development. For instance, while displaying high primary sequence homology to other ClpCs, *Mtb*ClpC1 displays unique mechanistic features. Indeed, contrary to its homologs, ClpC1 does not depend on activators as MecA and is fully competent to catalyze, together with ClpP1P2, the degradation of both unfolded (as casein) and folded (as GFPssra) substrates. In addition, *Mtb*ClpC1 is the sole target of the NPAs cyclomarin, lassomycin, ecumicin, or rufomycin.

In this study, we took advantage of a MD mutation (F444A) to stabilize the ClpC1 hexameric state. This mutation was chosen based on previous data obtained for *Sa*ClpC and our own results using *Mtb*ClpC1 that proved the existence of an equilibrium between a resting state and a hexameric form (7, 14). Indeed, the change of an aromatic phenylalanine to an alanine resulted in a large shift in the size exclusion profile towards the size expected for a hexameric complex.

While the introduction of the F444A mutation shifts the equilibrium towards the hexameric state, it may potentially modify *Mtb*ClpC1’s mechanism of action. For example, the first structure of a ClpC complex, the only one obtained using X-ray crystallography, was from an inactive mutant, unable to catalyze ATP hydrolysis and therefore protein degradation (20). We therefore biochemically characterized the F444A mutant with respect to ATPase activity and ClpP1P2-mediated protein degradation of unfolded and folded substrates and show that *Mtb*ClpC1_F444A_ is fully active, with increased specific activities *versus* the WT protein. This activation was not unexpected, considering that the equilibrium is shifted towards the active state, but it is worth mentioning that it is more moderate than the activation described for ClpC1 homologs with similar modifications in the MD (14). This fact likely reflects the functional differences between these homologs but nonetheless shows that the F444A mutant is fully able to catalyze unfolded and folded protein degradation in association with ClpP1P2.

One of our goals was to understand the mechanism of action of antibiotics targeting ClpC1. Therefore, an important question was whether the F444A mutation might influence the effects of cyclomarin and ecumicin. It is known that even though cyclomarin and ecumicin largely share their binding surfaces in the NTD, their binding results in different mechanistic consequences. In fact, as we have shown here and has been reported elsewhere (4, 5), ecumicin results in a marked increase in ClpC1 ATPase activity, whereas cyclomarin fails to do so. Two other striking differences concern FITC-casein and GFPssra degradation in association with ClpP1P2. Although both cyclomarin and ecumicin lead to a mild activation of FITC-casein degradation, ecumicin is a much better inhibitor of GFPssra degradation.

Introduction of the F444A mutation and the shift towards a hexameric state does not abolish cyclomarin and ecumicin effects, and a similar profile of activation and inhibition is observed for all enzymatic activities tested. This does not support the recent proposition, based on protein chimeras, that cyclomarin mechanism of action depends only on a shift of the resting state–hexamer equilibrium (16). If that was the case, the effect of cyclomarin should be abolished with the introduction of the mutant. Indeed, the same conclusion could be simply derived from the fact that the two antibiotic effects are dissimilar. If ecumicin and cyclomarin effects were exclusively dependent on resting state-hexamer equilibrium, we would expect their action to be the same in all activities measured—which is not the case.

The cryo-EM structures we determined further corroborate our assumption that the F444A mutant is a valid model for an active ClpC1 hexamer. Supporting the validity of our approach, multiple structural features demonstrate that we have in fact a functional enzyme. First, the asymmetric hexameric structure we observe here has been observed for other members of the AAA+ family, including a very recent structure of *Sa*ClpC as well as ClpA (21, 22). This shows that the mutation we introduced does not impair hexamer formation, domain architecture, or nucleotide binding. Even more importantly, the unexpected presence of a peptide bound in the pore formed by the D1 and D2 domains and in close contact with the canonical tyrosine loops demonstrates that F444A ClpC1 is fully competent to bind and translocate protein substrates.

In addition to the apo *Mtb*ClpC1 cryo-EM structure, we determined additional structures after the addition of either cyclomarin or ecumicin antibiotics to purified *Mtb*ClpC1. Both cyclomarin-bound *Mtb*ClpC1 as well as one of the two 3D classes of ecumicin-bound *Mtb*ClpC1 are strikingly similar to the apo *Mtb*ClpC1 structure and have missing NTDs, which contain the binding sites for both cyclomarin and ecumicin. The role of the NTD in ClpC1’s mechanism of action remains largely unknown, but data obtained with other AAA+ ATPases suggest that it may transfer the polypeptide substrate to D1 and the D1 pore. Curiously, truncating part of the ClpC1 NTD, residues 1 to 78, has been shown to paradoxically activate the refolding capability of ClpC1 (13). Considering our data, it seems, however, unlikely that cyclomarin would induce a stable conformational change in the NTD, as for example, occurs with MecA binding in other species, where an additional ring adjacent to the D1 ring is clearly observed both in X-ray and cryo-EM structures.

The second 3D class of ecumicin-bound *Mtb*ClpC1, containing roughly 40% of the imaged particles, reveals three globular domains above the D1 N-termini, most likely corresponding to three NTDs. Additional densities corresponding to the other NTDs are not observed, strongly suggesting that they remain flexible. Interestingly, the position of the putative NTDs in our map of ecumicin-bound *Mtb*ClpC1 is identical to the structure of an NTD trimer described for a hyperactive *Escherichia coli* ClpB MD mutant engaged with casein (26). In this structure, obtained at much higher resolution (2.9 Å), the NTD trimer forms a substrate entrance channel, positioning the casein polypeptide above the translocation pore. Additionally, trimerization of the NTD of *Mtb*ClpC1 upon addition of ecumicin was recently demonstrated in a crystal structure of the ecumicin–ClpC1_NTD_ complex (15). The relative position of the NTDs in the ecumicin: ClpC1_NTD_ trimer is similar to the NTD trimer in casein-bound *E. coli* ClpB and therefore fits equally well in our low-resolution map. Interestingly, contrary to the casein: ClpB_NTD_ trimer, the orientation of the individual NPA-binding sites of the NTDs in the ecumicin: ClpC1_NTD_ trimer is facing outwards rather than inwards. However, the lower resolution of the ecumicin-bound *Mtb*ClpC1 map we obtained here (8.6 Å) does not allow us to accurately assess the ecumicin-binding site or the relative conformations of the NTD domains.

How can the structures we obtained help in understanding the mechanism of action of cyclomarin and ecumicin? As described previously, albeit binding to similar regions of the NTD, cyclomarin and ecumicin result in different mechanistic consequences. Isothermal titration calorimetry results as well as X-ray crystallographic studies have shown that only one molecule of cyclomarin binds to the NTD (7, 11), forming a bridge over a hydrophobic ridge dominated by the aligned phenyl rings of phenylalanines F2 and F80. It is still unclear if cyclomarin, like rufomycin, forms a covalent adduct with the NTD N-terminal methionine. Quite striking is the fact that cyclomarin binding results in a dramatic increase in the stability of the NTD, and this is reflected in an increase of circa 15 °C in thermal stability as well as an important restriction of the domain dynamics induced by ArgP binding (7).

Ecumicin is a tridecamer depsipeptide with a larger scaffold than cyclomarin and an extended tail of three amino acids important for binding to the protein NTD (see Fig. S1). Furthermore, it has a different binding stoichiometry where two ecumicin molecules are accommodated by one NTD (15). Interestingly, in the crystal packing of the ecumicin:ClpC1_NTD_ complex, clusters of three dimers arrange themselves in a looser hexameric arrangement. Wolf *et al.* (15) suggested that the presence of two ecumicin molecules may help to stabilize this aggregate via hydrophobic binding. It is speculative to consider that a similar NTD trimer occurs in solution. Yet, the structure of ecumicin-bound ClpC1 shown here could represent such a complex, where ecumicin stabilizes the interaction between the three NTD subunits. Still, several mechanistical questions can be addressed here, particularly how the formation of an NTD trimer upon ecumicin binding can result in ATPase activation, inhibition of GFPssra degradation, and a mild activation of FITC-casein degradation. As described above, due to its high mobility, very little is known about NTD function. Nevertheless, two roles for the NTDs in ClpA have been proposed (27). One of the proposed roles is to work as an antenna to capture substrates via an initial weak interaction, transferring them to the second, stronger binding site for unfolding, and subsequent translocation to the D1-D2 pore. In this way, the NTDs would not be fundamental for catalysis but would make the processing of some challenging (folded) substrates more productive. This hypothesis is consistent with the observation that removal of half of the ClpC1 NTD appears not to affect firefly luciferase refolding by ClpC1 (28) or that casein degradation by ClpA (29) is independent of the NTD. In contrast to these unfolded substrates, impairment of the NTD can drastically affect the degradation of (folded) GFPssra by ClpA (30). Another putative role for the NTD would be to function as an “entropic brush” in order to prevent unspecific interactions with the pore-binding sites. Resulting from these two proposed roles, impairment of NTD function should likely affect the degradation of folded proteins like GFP more than unfolded proteins such as FITC-casein. The results presented here seem to corroborate this hypothesis, since a strong inhibition of GFPssra degradation and a mild activation of FITC-casein degradation by *Mtb*ClpC1 are observed after the addition of ecumicin. However, it is still not clear how the formation of NTD oligomers may explain the strong ATPase activation observed after the addition of ecumicin.

In summary, we provide here the first high-resolution structure of *Mtb*ClpC1, an important drug target, in its apo, cyclomarin-, and ecumicin-bound states. The ecumicin-bound ClpC1 structure presented here allows us to suggest a model for antibiotic action based on the formation of stable ecumicin-bound NTD oligomers, which would block folded substrate degradation by the ClpC1P1P2 complex.

## Experimental procedures

### Biochemistry

Mutations F444A, E288A, and E626A were introduced into a pet20-ClpC1 plasmid using an NZYMutagenesis kit. The primers used are listed in Table S1. Mutations were confirmed by DNA sequencing (Eurofins Scientific) using the T7 forward primer. ClpC1 and mutants were expressed and purified as previously described (7). ClpC1 ATPase activity and FITC-casein and GFPssra degradation by ClpC1P1P2 complex were measured as described previously (18). Differential scanning fluorimetry measurements were executed in a Pierce Light Cycler 96 using a 34 to 95 °C temperature ramp using 5 μM NTD, 12.5 μM cyclomarin and ecumicin, and 166 μM ArgP in Hepes pH 7.4 50 mM NaCl 100 mM. The T_m_ was calculated using the first derivate of the respective curves.

**Table:** 

Mutation	Forward primer	Reverse primer
F444A	5′GGCGGCCTTCTCGGCGTCCTGGGCGTCG 3′	5′-GGCGGCCTTCTCGGCGTCCTGGGCGTCG-3′
E288A	5′ATCCTGTTTATCGACGCGCTGCACACCTTGGTC-3′	5′-GACCAAGGTGTGCAGCGCGTCGATAAACAGGAT-3′
E626A	5′ GGTGCTGTTCGACGCGATCGAGAAGGCGC 3′	5′ GCGCCTTCTCGATCGCGTCGAACAGCACC 3′

### Cryo-EM sample preparation

Cryo-EM grids of Apo *Mtb* ClpC1 (*Mt*ClpC1), *Mt*ClpC1 in complex with cyclomarin, and *Mt*ClpC1 in complex with ecumicin were vitrified using a Vitrobot Mark IV (FEI). Quantifoil Cu/Rh 1.2/1.3 300 mesh grids were previously glow-discharged for 30 s at 15 mA. Aliquots of 3 μl of the different samples were added onto the grids, blotted for 3 s at 4 °C and 95% humidity, and plunged into liquid ethane.

### Cryo-EM data collection

Screening and data acquisition of all samples were performed using a 200 kV FEI Talos Arctica equipped with a Falcon III direct electron detector at the Centro Nacional de Biotecnología cryo-EM facility. A total of 944 movies of Apo *Mt*ClpC1, 2840 movies of *Mt*ClpC1 + cyclomarin, and 1520 movies of *Mt*ClpC1 + ecumicin were acquired at a nominal magnification of 120,000× (corresponding to a pixel size of 0.855 Å/pixel), with a defocus range of −1.2 to −3.1 μm. Movies were fractionated to 60 frames with a total exposure of 40 s (Apo *Mt*ClpC1 and *Mt*ClpC1 + ecumicin) or 30 s (*Mt*ClpC1 + cyclomarin), with a total dose per movie of 34.3 e^−^/Å^2^ (Apo *Mt*ClpC1), 36.9 e^−^/Å^2^ (*Mt*ClpC1 + cyclomarin), and 32.2 e^−^/Å^2^ (*Mt*ClpC1 + ecumicin).

### Cryo-EM data processing

Collected movies (Apo dataset: 944, cyclomarin-bound dataset: 2840, ecumicin-bound dataset: 1520) were motion corrected and dose weighted using MotionCor2 (31). Further data processing was performed using cryoSPARC 3.3.1 (27). Initial CTF estimation on the imported aligned and dose weighted micrographs was performed using Patch CTF estimation. For each dataset, particle picking was performed using crYOLO (32). Imported particle stacks were extracted using a box size of 500 pixels downsampled to 250 pixels (2× binned), corresponding to a pixel size of 1.71 Å/pixel. Extracted particle stacks were cleaned using several rounds of iterative 2D classification and 2D class selection. Cleaned particle stacks were used as an input for *Ab Initio* model generation and subsequent NU 3D refinement. For Apo and cyclomarin-bound datasets, a final NU 3D refinement was performed on unbinned particles from the prefinal 3D refinement, extracted using a box size of 500 pixels, corresponding to a pixel size of 0.885 Å/pixel. For the Apo dataset, the final NU refinement was performed using optimized per-group CTF parameters, whereas for the cyclomarin-bound dataset, both per-group CTF parameters as well as per-particle defocus were optimized. For the ecumicin-bound dataset, particles selected after 2D classification were used as an input for *Ab Initio* model generation and hetero refinement using two classes, and each class was separately refined using a final NU 3D refinement. Maps for the apo and cyclomarin-bound datasets as well as class 1 of the ecumicin-bound dataset were postprocessed using DeepEMhancer (https://github.com/rsanchezgarc/deepEMhancer) for model building purposes (33).

### Model building and refinement

An initial model was generated based on the AlphaFold2 (34) prediction for monomeric ClpC1 (https://alphafold.ebi.ac.uk/entry/P9WPC8). First, parts with a low per-residue confidence score were removed from the AlphaFold2 ClpC1 monomer model. Next, six copies of the trimmed ClpC1 model were manually placed in the DeepEMhancer-sharpened Apo ClpC1 map using rigid-body fitting in USCF Chimera (35), followed by automatic molecular dynamics–based flexible fitting using NAMDINATOR (36) and subsequent manual building in Coot (37). The resulting structure was further refined in the cryoSPARC-sharpened Apo ClpC1 map in Phenix, using global minimization, local grid search, ADP refinement, secondary structure and Ramachandran restraints, noncrystallographic symmetry constraints, and a nonbonded weight parameter of 300.

The apo structure of *Mtb* ClpC1 was subsequently placed in the DeepEMhancer-sharpened ClpC1 + cyclomarin and ClpC1 + ecumicin, class 1 maps using rigid-body fitting in UCSF Chimera, followed by manual building in Coot. Next, several cycles of refinement using the cryoSPARC-sharpened maps were performed in Phenix, using global minimization, local grid search, ADP refinement, secondary structure and Ramachandran restraints, noncrystallographic symmetry constraints, and a nonbonded weight parameter of 300.

For ClpC1 + ecumicin class 2, the ClpC1 + ecumicin class 1 structure was rigid-body fitted in the map using UCSF Chimera, together with three copies of an available structure of the *Mtb* ClpC1 NTD in complex with Ecumicin PDB-ID: 6PBS (15), aligned with the *E. coli* ClpB NTD trimer found in PDB-ID 6OG3 (26).

## Data availability

Atomic coordinates and associated structure factors have been deposited in the PDBs 8AUA, 8AUV, and 8AUW.

## Supporting information

This article contains supporting information.

## Conflict of interest

The authors declare that they have no conflicts of interest with the contents of this article.
