# Supplementary material for: Structure of the drug target ClpC1 unfoldase in action provides insights on antibiotic mechanism of action
Source: J Biol Chem. 2022 Oct 6;298(11):102553. doi: 10.1016/j.jbc.2022.102553 (PMC9661721; doi:10.1016/j.jbc.2022.102553)
Supplement: Supplemental Table S1 [file mmc1.pdf]

Supplementary Table 1. Cryo-EM data collection, processing, refinement and validation statistics

	*Mtb*ClpC1 Apo	*Mtb*ClpC1 + Cyclomarin	*Mtb*ClpC1 + Ecumicin, Class 1	*Mtb*ClpC1 + Ecumicin, Class 2
	EMD-15240<br>PDB 8A8U	EMD-15241<br>PDB 8A8V	EMD-15242<br>PDB 8A8W	EMD-15243
**Data collection and processing**				
Magnification	120,000	120,000	120,000	120,000
Voltage (kV)	200	200	200	200
Electron exposure (e–/Å^2)	34.3	36.9	32.2	32.2
Defocus range (μm)	1.2 – 3.1	1.2 – 3.1	1.2 – 3.1	1.2 – 3.1
Pixel size (Å)	0.885	0.885	1.71<br>(2x binned)	1.71<br>(2x binned)
Symmetry imposed	C1	C1	C1	C1
Initial particle images (no.)	264,417	516,615	336,885	336,885
Final particle images (no.)	66,564	102,018	45,698	30,976
Map resolution (Å)	3.62	3.34	4.29	8.59
FSC threshold	0.143	0.143	0.143	0.143
**Refinement**				
Model resolution, masked (Å)	3.9	3.6	4.9	N.A.
FSC threshold	0.5	0.5	0.5	
Map sharpening *B* factor (Å^2)	-114,1	-116.5	-164.7	-545.6
Model composition				
Non-hydrogen atoms	27,129	27,164	27,187	N.A
Protein residues	3414	3419	3423	
Water				
Ligands (ADP)	10	10	10	
B factors (Å^{-2})				
Protein	109.48	62.00	184.61	N.A.
Ligand	76.56	37.08	139.20	
R.m.s. deviations				
Bond lengths (Å)	0.006	0.005	0.004	N.A
Bond angles (°)	1.162	1.103	1.115	
Validation				
MolProbity score	1.66	1.67	1.66	N.A
Clashscore	8.01	8.11	9.63	
Poor rotamers (%)	0.04	0.11	0.00	
CaBLAM outliers (%)	1.51	1.39	1.51	
CC (mask)	0.76	0.79	0.80	
EMRinger	1.16	1.81	0.45	
Ramachandran plot				
Favored (%)	96.55	96.47	97.18	N.A.
Allowed (%)	3.45	3.53	2.82	
Outliers (%)	0.00	0.00	0.00	
Ramachandran-Z score				
Whole	-0.41	-0.60	-0.26	
Helix	-0.17	-0.33	-0.13	
Sheet	0.35	-0.44	-0.01	
Loop	-0.07	-0.16	-0.01	

Table 1 Cryo-EM data collection, processing, refinement and validation statistics
