# Supplementary material for: Structure of the drug target ClpC1 unfoldase in action provides insights on antibiotic mechanism of action
Source: J Biol Chem. 2022 Oct 6;298(11):102553. doi: 10.1016/j.jbc.2022.102553 (PMC9661721; doi:10.1016/j.jbc.2022.102553)
Supplement: Supplemental Figure S1 [file mmc2.pdf]

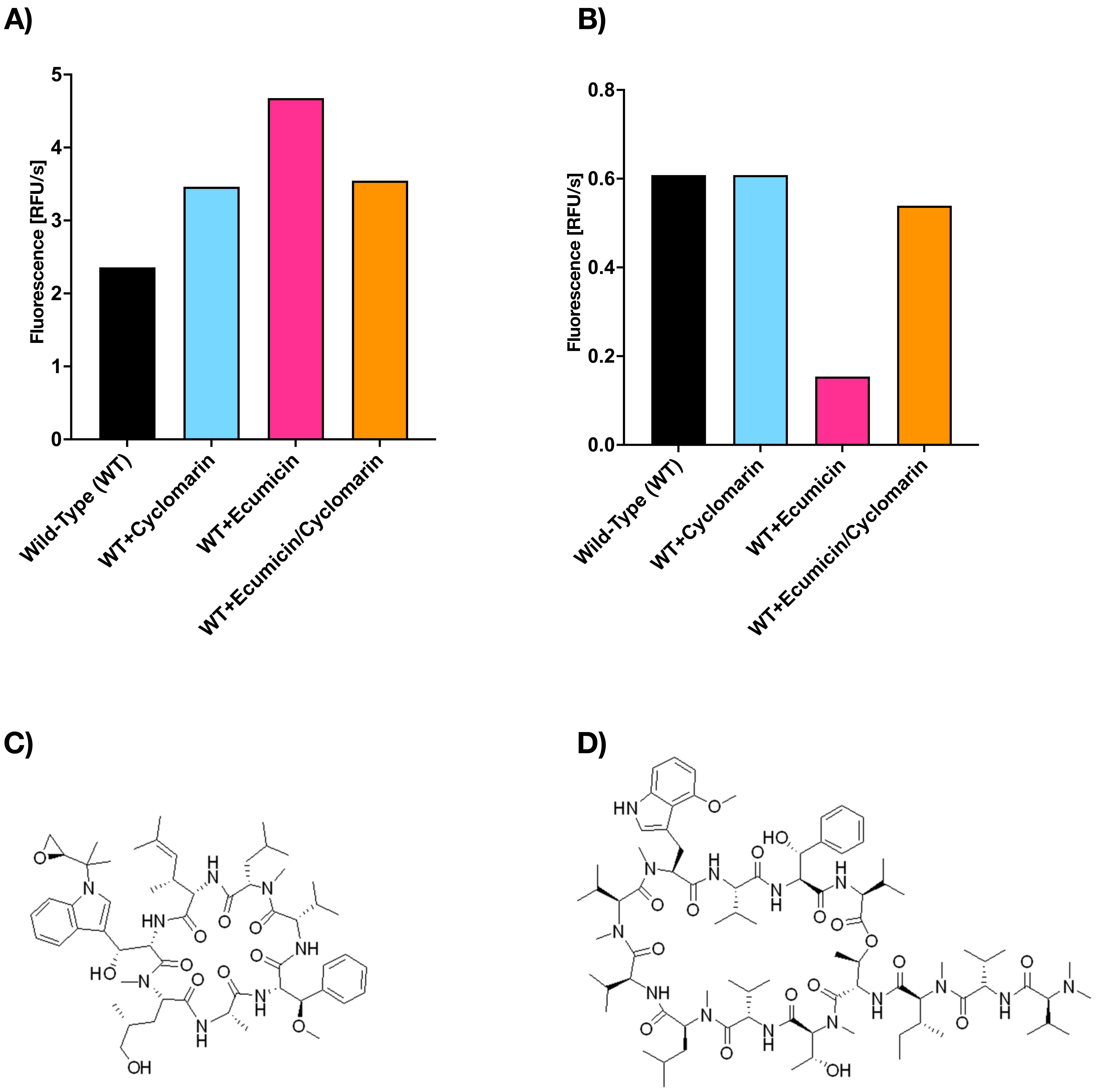

Supplementary Figure 1 Functional data

FITC casein (**A**) and GFPssra (**B**) degradation of *Mtb* ClpC1P1P2 with either one NPA (Cyclomarin or Ecumicin) or both NPAs bound. Structures of the natural product antibiotics Cyclomarin (**C**) and Ecumicin (**D**).
