# Supplementary material for: Structure of the drug target ClpC1 unfoldase in action provides insights on antibiotic mechanism of action
Source: J Biol Chem. 2022 Oct 6;298(11):102553. doi: 10.1016/j.jbc.2022.102553 (PMC9661721; doi:10.1016/j.jbc.2022.102553)
Supplement: Supplemental Figure S2 [file mmc3.pdf]

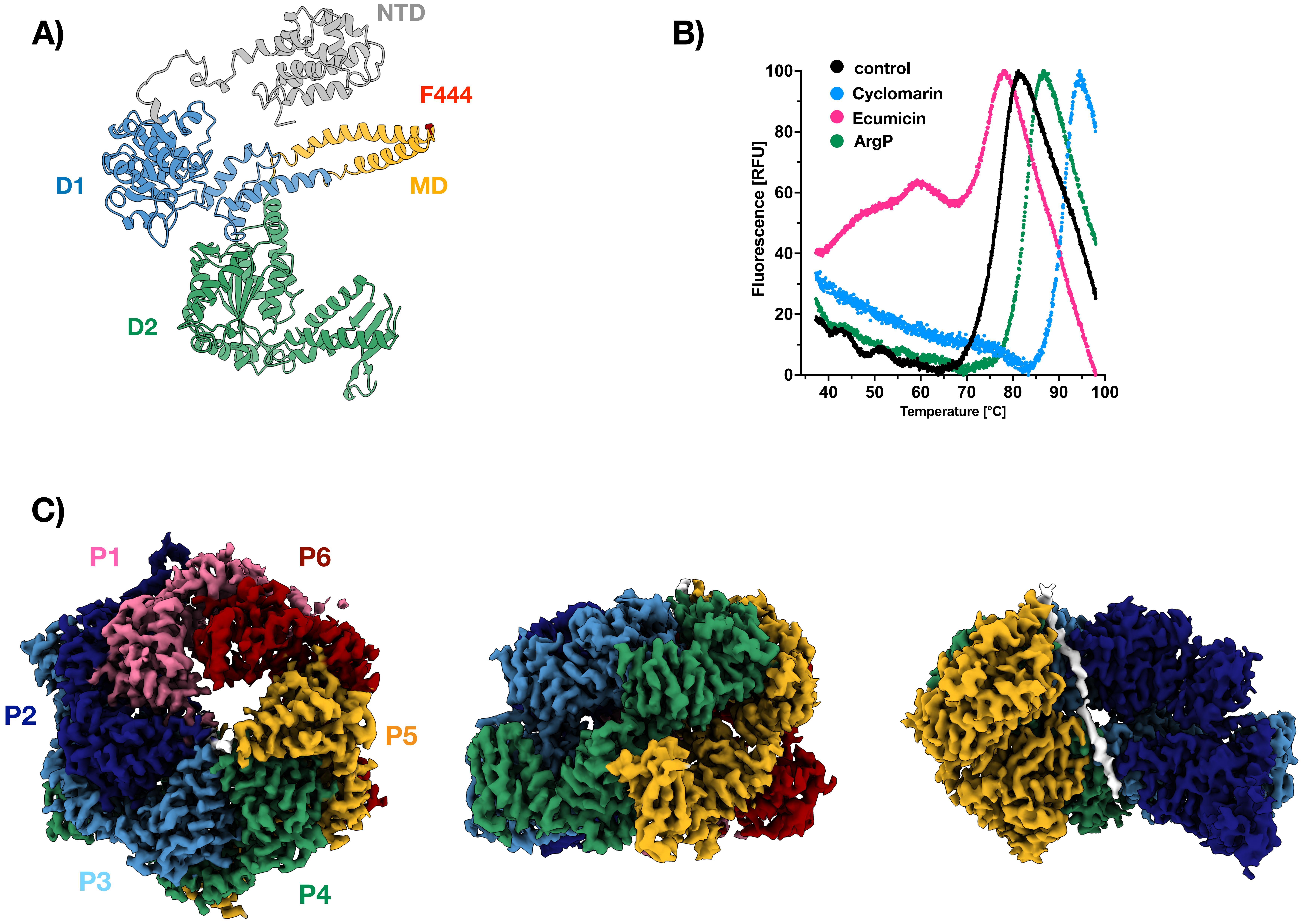

Supplementary Figure 2 ClpC1 domains and the hexameric structure of Cyclomarin bound ClpC1

A) Swiss-model of the ClpC1 monomer based on the structure of *B. subtilis* ClpC (PDB: 3PXI). The NTD domain is shown in grey, the MD in orange, the D1 in blue and the D2 domain in green. Residue F444 is marked in red. **B)** Differential scanning fluorometry curves of the ClpC1 NTD bound to natural product antibiotics and the substrate mimic Arginine-phosphate (ArgP). **C)** Cryo-EM map of Cyclomarin bound *Mtb*ClpC1 in top, side and substrate visible view. Protomers are coloured individually and labelled P1-P6, with P1 at the lowest bound position with respect to the substrate.
