# Supplementary material for: Structure of the drug target ClpC1 unfoldase in action provides insights on antibiotic mechanism of action
Source: J Biol Chem. 2022 Oct 6;298(11):102553. doi: 10.1016/j.jbc.2022.102553 (PMC9661721; doi:10.1016/j.jbc.2022.102553)
Supplement: Supplemental Figure S3 [file mmc4.pdf]

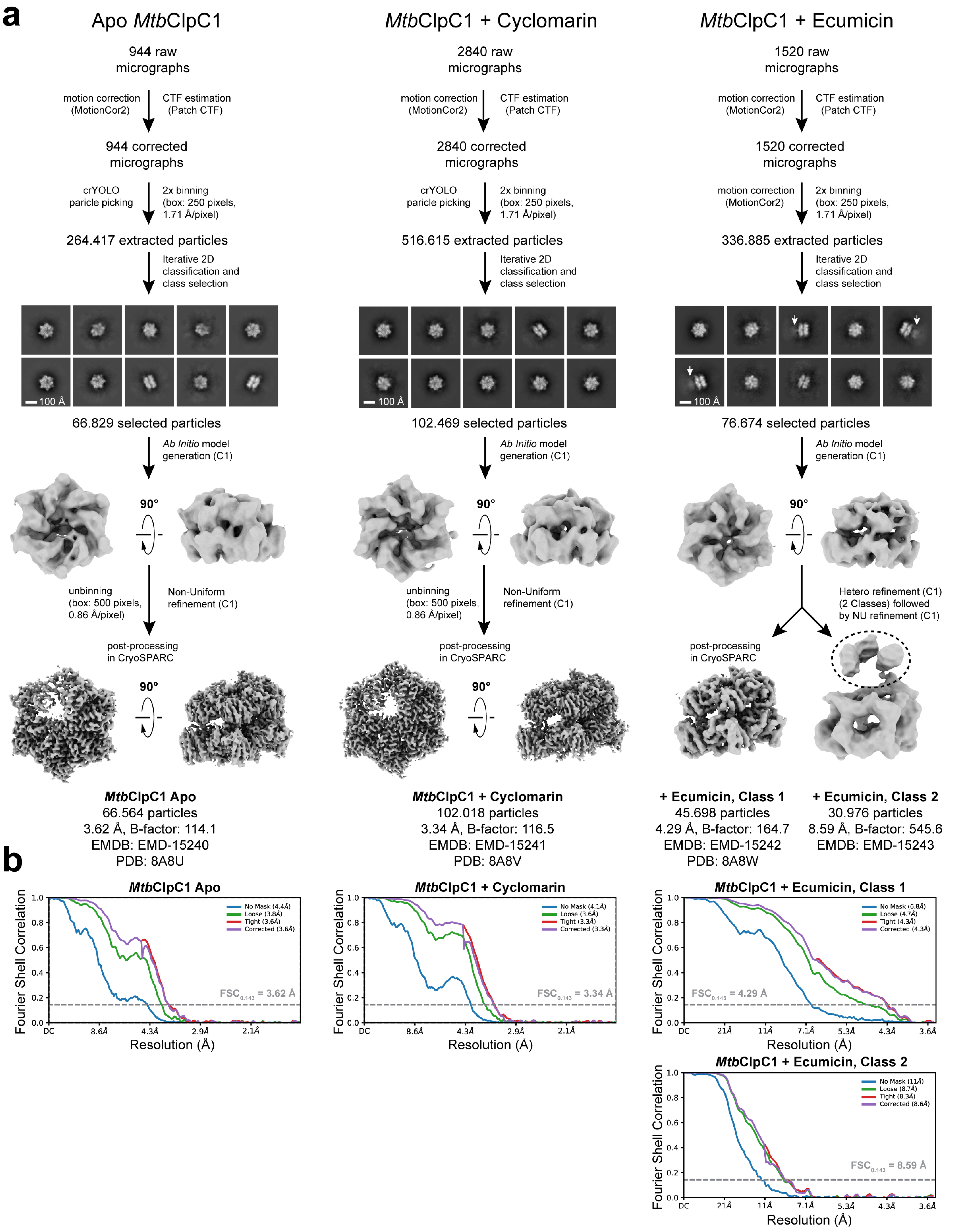

Supplementary Figure 3, cryo-EM data processing flow chart.

A) Processing workflow for Apo *Mtb*ClpC1, *Mtb*ClpC1 + Cyclomarin, and *Mtb*ClpC1 + Ecumicin structure determination by single particle analysis (SPA). Software packages used during processing of the individual datasets are indicated. **B**) Gold-standard FSC curves for Apo *Mtb*ClpC1, *Mtb*ClpC1 + Cyclomarin, and *Mtb*ClpC1 + Ecumicin Class 1 and 2. FSC curves are displayed after applying no mask (blue), a loose mask (green), a tight mask (red), or a tight mask with correction by noise substitution (purple) to both half maps before calculating FSC curves. The resolution at FSC = 0.143 is displayed for the corrected FSC curves (purple) as a dotted grey line.
