# Supplementary material for: Structure of the drug target ClpC1 unfoldase in action provides insights on antibiotic mechanism of action
Source: J Biol Chem. 2022 Oct 6;298(11):102553. doi: 10.1016/j.jbc.2022.102553 (PMC9661721; doi:10.1016/j.jbc.2022.102553)
Supplement: Supplemental Figure S4 [file mmc5.pdf]

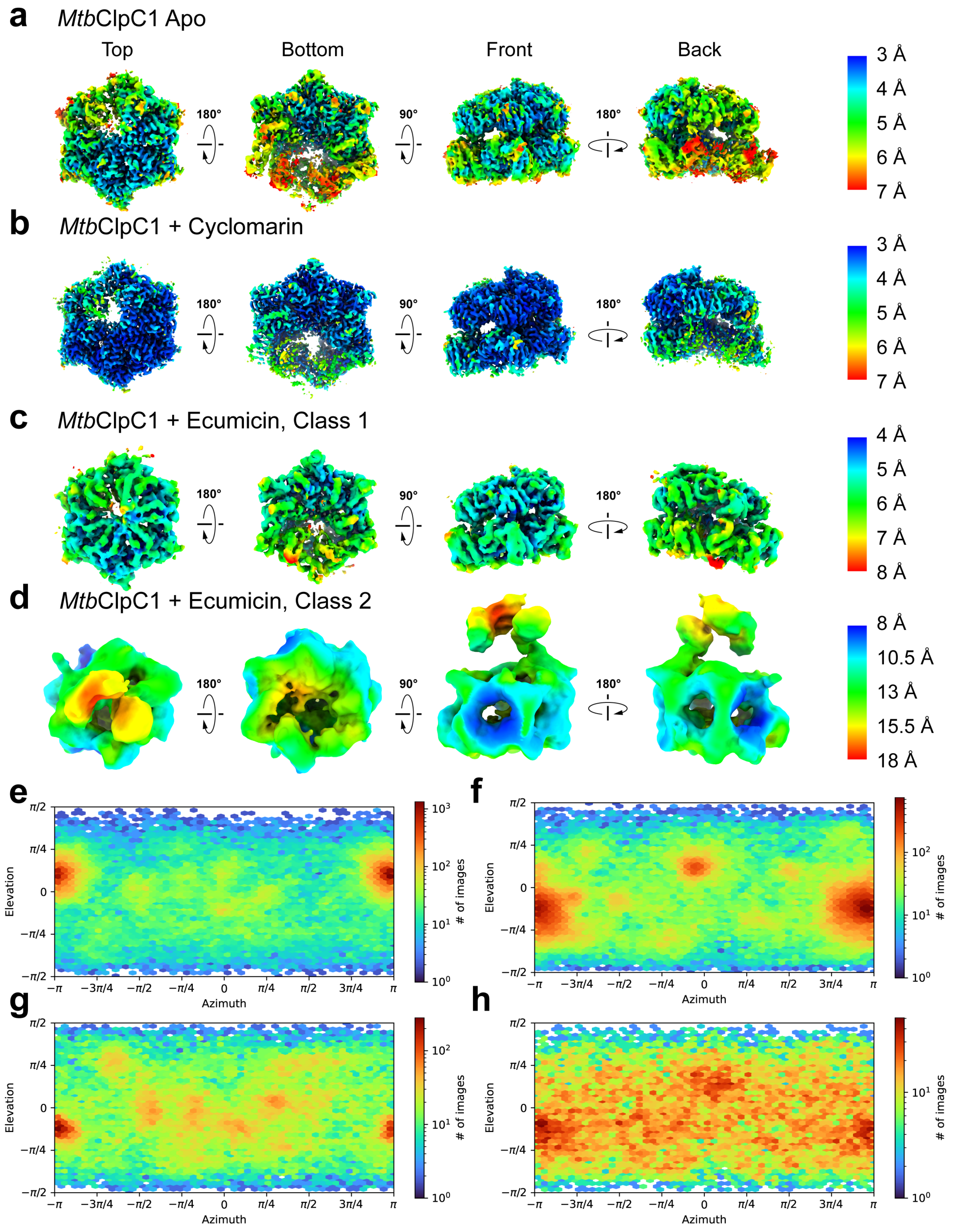

Supplementary Figure 4, Local resolution estimation and particle distribution of 3D reconstructions for Apo *Mtb*ClpC1, *Mtb*ClpC1 + Cyclomarin, and *Mtb*ClpC1 + Ecumicin Class 1 and 2.

Maps colored to local resolution are shown for Apo *Mtb*ClpC1 (**A**), *Mtb*ClpC1 + Cyclomarin (**B**), and *Mtb*ClpC1 + Ecumicin Class 1 (**C**) and *Mtb*ClpC1 + Ecumicin Class 2 (**D**). Plots displaying the distribution of particle orientations are shown for Apo *Mtb*ClpC1 (**E**), *Mtb*ClpC1 + Cyclomarin (**F**), and *Mtb*ClpC1 + Ecumicin Class 1 (**G**) and *Mtb*ClpC1 + Ecumicin Class 2 (**H**).
